# Three-Dimensional Porous PVDF Foam Imprinted Membranes with High Flux and Selectivity toward Artemisinin/Artemether

**DOI:** 10.3390/molecules28217452

**Published:** 2023-11-06

**Authors:** Weibai Bian, Ruixuan Zhang, Xiaohui Chen, Chuanxun Zhang, Minjia Meng

**Affiliations:** 1 School of Photoelectric Engineering, Changzhou Institute of Technology, Changzhou 213032, China; 21230532@czu.cn (R.Z.); chenxh@czu.cn (X.C.); 2 School of Chemistry and Chemical Engineering, Jiangsu University, Zhenjiang 212013, China; zcx622820@163.com; 3 Tianhe Pharmaceutical Co., Ltd., Yangzhou 225267, China

**Keywords:** foam imprinted membranes, selective separation, high flux, artemisinin

## Abstract

In this study, a new 3D porous PVDF-foam-imprinted membrane (PPIM) for the selective separation of artemisinin (ART) was first prepared via the dopamine adhesion of pre-synthesized MIPs into the interior of the PPIM. In the PPIM, the pre-synthesized molecularly imprinted polymers (MIPs) with artesunate (ARU) as a dummy template were uniformly loaded on the interior of the membrane, avoiding the defects of recognition site encapsulation found in the conventional membrane. This membrane also exhibited excellent flux, which is beneficial in practical separation applications. The PPIM was systematically characterized via FT-IR, SEM, pore-size distribution analysis, water contact angle test, membrane flux, and mechanical performance analysis, respectively. In the static adsorption experiment, the pseudo-second-order kinetic model better fitted the rebinding data of ART. Under dynamic conditions, the ART adsorption capacity of the PPIM could be further remarkably improved by tailoring the flow rate to 3 mL min^−1^. In the selective separation experiment, with artemether (ARE) as the competition substrate, the selective separation ability (*α*) of the PPIM towards ART/artemether (ARE) reached its peak value (3.16) within only 10 min at this flow rate, which is higher than that of porous PVDF foam non-imprinted membranes (PPNM) (ca. 1.5), showing great separation efficiency in a short time. Moreover, the PPIM can be reused five times without a significant decrease in its adsorption capacities, showing good regeneration performance. This work highlights a simple strategy for constructing new MIMs with high flux and great mechanical strength to achieve the efficient selective separation of ART and ARE in practical applications.

## 1. Introduction

Artemisinin (ART) is an effective and widely used drug for the treatment of malaria [1,2]. Among the ether derivatives of artemisinin, artemether (ARE) has the highest antimalarial activity—six times that of artemisinin [3,4]. It also has the advantage of being fast-acting with low toxicity compared to artemisinin, has good oil solubility, which makes it easier to be converted into other biological agents, and has high therapeutic value. The intramuscular injection of artemether was once used as an important treatment for malaria. 

In existing synthesis technology, artesunate has always been obtained via the reduction of artemisinin into dihydroartemisinin using sodium borohydride in methanol or ethanol as the solvent, finally followed by etherification [5]. Therefore, the artemether currently available in the market is often obtained via methylation using artemisinin as a raw material. However, in the synthesis process of preparing artemether from artemisinin as a reactant, there is always some unreacted artemisinin within the raw material, which leads to a decrease in artemether purity. The current application of artemether in pharmacology has certain safety impacts on human health [6]. Therefore, it is important to improve the purity of the artemether during the production process.

The molecular imprinting technique (MIT) is a method used for the specific recognition of target molecules by simulating “antigen” and “antibody” interactions [7,8]. The main principle is to form a complex by pre-polymerizing a template molecule (target molecule, imprinted molecule) with a selected functional monomer molecule using various forces (covalent bonding, coordination, hydrogen bonding, electrostatic force, hydrophobic interaction, etc.), and then an appropriate amount of specific cross-linking agent is added to the complex mixture, forming a highly cross-linked polymer using an initiator light or thermal polymerization. Finally, the imprinted molecules are eluted from the polymer to form a molecularly imprinted polymer (MIP) [7,8,9,10,11]. This gives the polymer an amazing “memory” function and a very selective adsorption ability towards the target molecule. Molecularly imprinted membrane technology [12] is a new crossover technology that combines the advantages of specificity and the high selectivity of MIT with the advantages of easy and continuous operation and mild conditions of membrane separation technology, and it is widely used in the fields of the separation and purification of substances, bionic sensors, and solid phase extraction [13,14]. The common preparation methods of molecularly imprinted membranes (MIMs) are mainly interfacial condensation, surface grafting, electrostatic spinning, and phase conversion techniques, among which phase conversion is the most commonly used method to prepare molecularly imprinted membranes. However, traditional molecularly imprinted membranes for phase conversion are prone to swelling and cause the encapsulation of imprinted sites in the recognition sites, resulting in decreased selectivity [15,16]. Moreover, MIMs generally have rather low membrane fluxes and large mass transfer resistance [17,18,19]. Therefore, it is important to explore new MIMs with high flux and high selectivity to solve the flux/perm-selectivity trade-off phenomenon for future industrial applications.

Foam-type membranes with three-dimensional (3D) cross-network structures may provide an ideal membrane substrate that can provide sufficient space for MIP loading and high flux with abundant pores. Li constructed a PVDF frame (CoFe LDH(F)/PVDF) with the NH_4_F-assisted hydrothermal growth of CoFe LDH(F)/PVDF nanoarrays to achieve an ultra-fast decolorization effect in flowing dye wastewater with high flux [20]. As we know, the adhesive proteins of mussels contain unusually high concentrations of catechol and amine functional groups, which are capable of mediating adhesion to most organic and inorganic surfaces. Messersmith’s group first reported that polydopamine (PDA) was able to mimic the adhesive proteins of mussels, and various surfaces were deposited with these bioinspired polymers simply via the oxidative polymerization of dopamine in alkaline conditions [21,22,23]. Inspired by this breakthrough, as well as several successful examples of using PDA in molecular imprinting [24,25], we intend to use PDA as a bridge to introduce the MIP particles to foam-type membranes to construct a 3D MIM with high flux.

In this study, we introduced the synthesized MIPs into the interior of the foam PVDF membrane using dopamine adhesion to obtain a large-throughput foam PVDF/PDA-based imprinted membrane (PPIM) that avoids the defects of recognition site encapsulation. It is noteworthy to mention that the preparation of MIPs relied on the dummy template approach with its analog artesunate (ARU) as the real template. This choice was made because the ART molecule lacks active groups such as –OH, –COOH, and –NH_2_ that can engage with the functional monomers. On the other hand, in consideration of the structure of ARU, like ART and the extra carboxyl as an effective active group, the ARU molecule was selected as the template molecule. The MIP was prepared via precipitation polymerization as follows: acrylamide (AM) was used as the functional monomer, artesunate (ARU) as the dummy template molecule, ethylene glycol di-methacrylate (EGDMA) as the crosslinker, and 2,2′-azobis(2-methylpropionitrile) (AIBN) as the initiator. The preparation and characteristics of the PPIM and the non-imprinted membrane (PPNM) were investigated, and then the adsorption properties of the PPIM toward ART were explored. In addition to static adsorption, the PPIM can also be used as a dynamic adsorption membrane to evaluate the dynamic adsorption behavior and selectivity of the material.

1:8 resulted in large holes in the membrane due to the excessive number of NaCl particles added, which led to the membrane becoming too fragile. Therefore, the mass ratio of 1:7 was chosen as the optimal ratio of PVDF powder/sodium chloride particles for preparing the foam PVDF membrane.

## 2. Results and Discussion

### 2.1. Optimization of the Reactant Ratio of Foam PVDF Membrane

The experiment optimized the dosage of PVDF powder and sodium chloride particles (the mass ratios were 1:6, 1:7, and 1:8), and three different foam PVDF membranes were prepared, as shown in Figure S2. After the synthesis, it was found that the PVDF membrane was easy to break when the mass ratios of PVDF powder/sodium chloride increased to 1:8, which is due to the excessive pores formed by the elution of sodium chloride. Although the foam PVDF membranes with mass ratios of 1:6 and 1:7 both showed good appearance, the insufficient addition of sodium chloride particles (1:6) directly led to fewer pores in the prepared foam PVDF membrane, which does not favor more MIP loading inside the membrane. On the contrary, the foam PVDF membrane with a ratio of 1:8 resulted in large holes in the membrane due to the excessive number of NaCl particles added, which led to the membrane becoming too fragile. Therefore, the mass ratio of 1:7 was chosen as the optimal ratio of PVDF powder/sodium chloride particles for preparing the foam PVDF membrane.

### 2.2. Morphology Analysis

**Figure 1:**
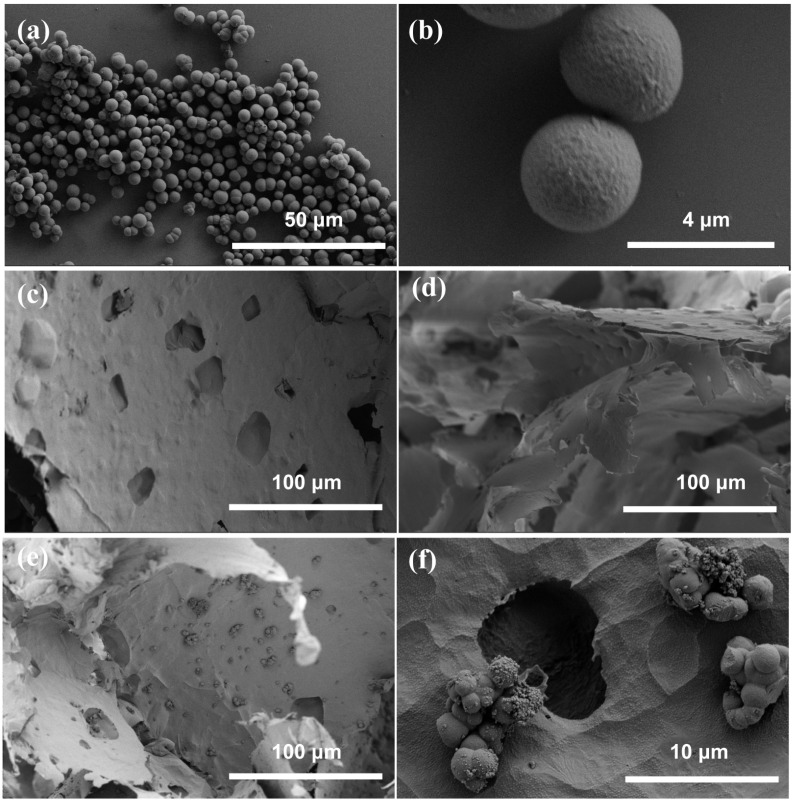
SEM images of MIPs (**a**,**b**), the cross section image of foam PVDF membrane (**c**,**d**), the cross section image of PPIM (**e**,**f**) with the different magnifications.

SEM images of the MIPs, foam PVDF membrane, and PPIM are shown in Figure 1. As shown in Figure 1a,b, the MIPs with particle sizes of ca. 2.0~3.5 mm were successfully synthesized. Then, it could be clearly seen that the surface of the cross section of the foam PVDF membrane was smooth. Figure 1e,f show that after the MIPs were introduced into the foam PVDF membrane via PDA adhesion, they were successfully introduced into the surface (Figure S3) and interior of the PPIM. Figure S3 shows the SEM surface image of the PPIM and PPNM with the same phenomenon, indicating that these membranes had been successfully prepared.

### 2.3. Membrane Pore Size Analysis

**Figure 2:**
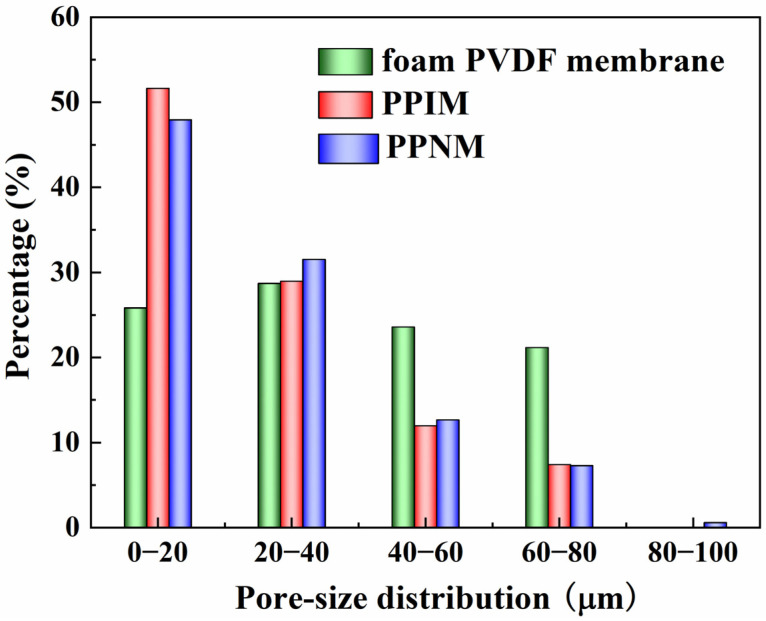
Pore–size distribution of foam PVDF membrane, PPIM and PPNM.

The pore size distribution of the foam PVDF membrane, PPIM, and PPNM are shown in Figure 2. It can be seen that the pore size of the foam PVDF membrane was mainly distributed between 0 and 80 μm. After the PDA adhesion of the foam PVDF membrane and MIPs/NIPs, the pore size became significantly smaller, mainly being distributed in the range of 0–40 μm, as shown in Figure 2. This change can be explained as the successful loading of MIPs/NIPs into the pores of the interior of the foam PVDF membrane and occupying the space of the membrane, which also shows that the PPIM was successfully synthesized. Additionally, the pore size distribution of the PPNM was similar to that of the PPIM, which was due to the same composition of MIPs and NIPs loaded on the foam PVDF membrane.

### 2.4. FT-IR Analysis

**Figure 3:**
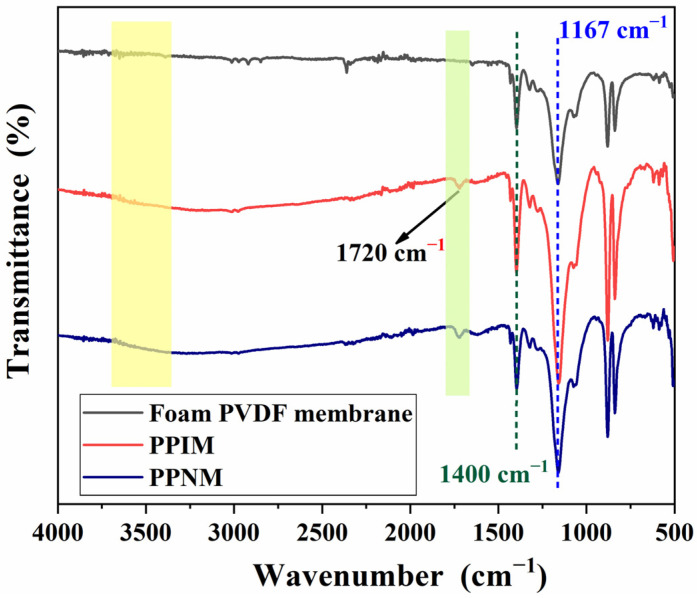
FT–IR spectra of foam PVDF membrane, PPIM and PPNM.

Figure 3 shows the FT-IR spectroscopy of the foam PVDF membrane, PPIM, and PPNM. As shown in the figure, the characteristic peak structure appears at 1400 cm^−1^ and 1167 cm^−1^ attributed to the C-F vibration in the foam PVDF membrane. Compared with the foam PVDF membrane, the new characteristic peak at 1720 cm^−1^ appeared in the PPIM and PPNM, which may be ascribed to the stretching vibration of the carboxyl group (C=O) of the MIPs in the membrane (PPIM/PPNM). It was also found that there was a characteristic broad peak at around 3500 cm^−1^ in the PPIM and PPNM. This may be due to the stretching vibration of the hydroxyl group of the PDA molecule, indicating the successful modification of PDA to the PPIM. In addition, due to the same chemical composition after removing the template, there is almost no difference in the spectra between the PPIM and PPNM.

### 2.5. Wettability Analysis

**Figure 4:**
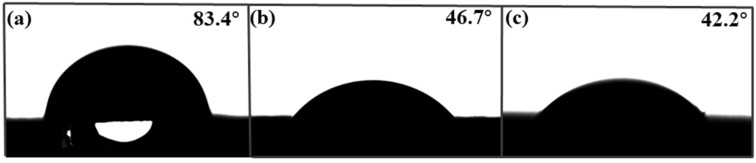
The contact angle of foam PVDF membrane (**a**), PPIM (**b**), and PPNM (**c**).

Contact angle (CA) is an important factor affecting adsorption performance. Figure 4 shows the contact angle images of the foam PVDF membrane, PPIM, and PPNM. We can see that the contact angles of the foam PVDF membrane was 83.4°. As we know, pure PVDF powder should be hydrophobic, but there were rich pores in the surface and interior of the foam PVDF membrane. Thus, the contact angle of the foam PVDF membrane was less than 90° due to the water droplets penetrating directly through the pores in the membrane. The corresponding contact angles of the PPIM and PPNM were 46.7° and 42.2°, indicating that the hydrophilicity increased after the PDA/MIP modification. This was due to there being many polar groups of hydroxyl groups and amino groups in the introduction of PDA in the PPIM and PPNM. 

### 2.6. Membrane Flux Studies

**Figure 5:**
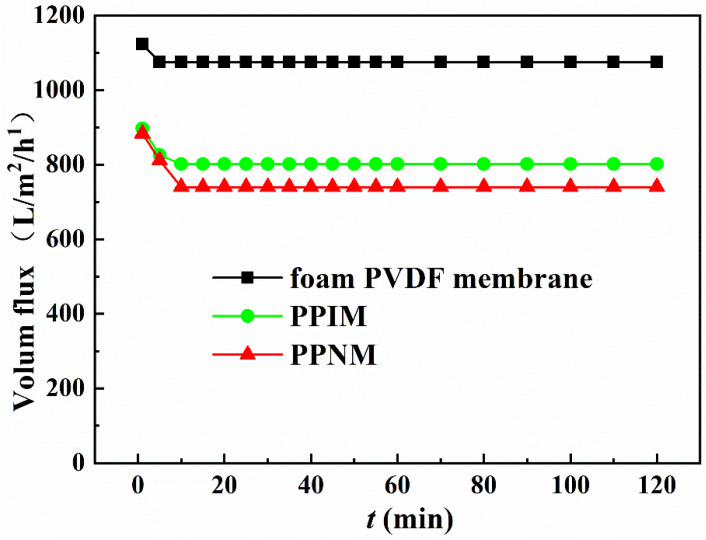
The flux of ethanol solution through different membranes.

Flux is an important condition that affects membrane separation capability. Considering that the subsequent adsorption experiments were all carried out in an ethanol solution, here, the ethanol flux of the foam PVDF membrane, PPIM, and PPNM were tested under a pressure of 10 Kpa. As shown in Figure 5, the fluxes of the foam PVDF membrane, PPIM, and PPNM were large at the initial moment, about 1122.61 L/m^2^/h, 897.13 L/m^2^/h, and 883.24 L/m^2^/h, respectively. After that, the flux decreased over time and basically reached a stable level in about 10 min. In addition, compared with foam PVDF membrane, the fluxes of PPIM and PPNM were significantly reduced. This was possibly ascribed to the MIPs and NIPs introduced by PDA occupying the holes of the membrane, which hindered the passage of ethanol to a certain extent. Nevertheless, PPIM and PPNM still kept larger fluxes with 801.59 L/m^2^/h and 789.93 L/m^2^/h, respectively, which indicated that the PPIM and PPNM can achieve good separation requirements for a long time in practical applications. 

### 2.7. Mechanical Performance Analysis

**Figure 6:**
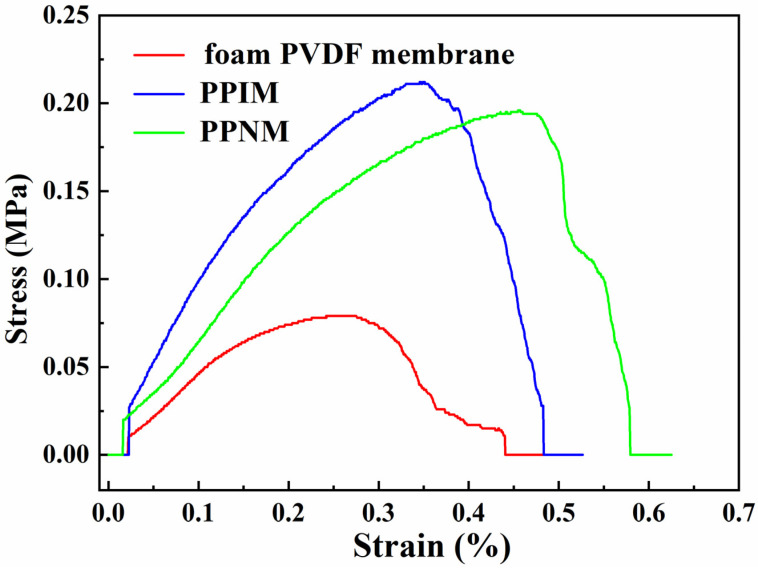
Tensile performance curves of foam PVDF membrane, PPIM and PPNM.

Mechanical strength is one of the important effects of the membrane in practical applications. Therefore, we measured the stress–strain curve to quantitatively study the mechanical properties of the membranes. Figure 6 shows the tensile stress curves of the PPIM, PPNM, and foam PVDF membrane. It can be seen the tensile stress and elongation at break of the foam PVDF membrane were relatively low, being 0.077 MPa and 0.44%, respectively. In contrast, the mechanical properties of the PPIM and PPNM prepared in this experiment were significantly improved, with the tensile stress and elongation at break increasing more than twice, to 0.20 MPa and 0.48%, respectively. The corresponding tensile stress and elongation at break for the PPNM is about 0.19 MPa and 0.57%. It was obvious that the tensile properties of the PPIM and PPNM were better than those of the foam PVDF membrane, which was mainly due to the introduction of PDA in the membrane, improving its mechanical strength.

### 2.8. Static Adsorption Experiment

**Figure 7:**
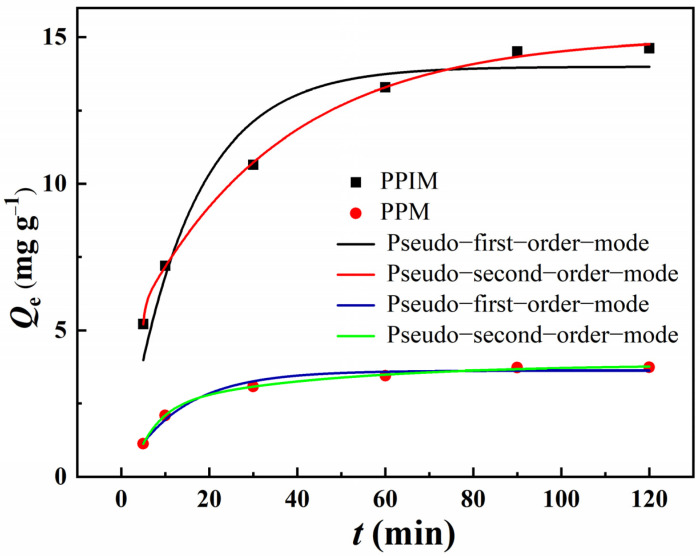
Adsorption kinetic curves and fitting model of PPIM and PPNM.

The adsorption kinetic curves of ART on the PPIM and PPNM are shown in Figure 7. It can be seen from the figure that, due to the large number of imprinted sites loaded on the PPIM, the adsorption capacity toward ART increased rapidly in the first 30 min, and then the imprinting sites were occupied and the adsorption gradually reached equilibrium. The adsorption trend of the PPNM was also the same, but the equilibrium adsorption capacity was lower than that of the PPIM, because there were no effective imprinting sites on the membrane. The kinetic data were analyzed through the pseudo-first-order equation and the pseudo-second-order equation.

The pseudo-first-order model is depicted as follows [26]:

(1) $$Q_{t}=Q_{e}-Q_{e}e^{-k_{1}t}$$

The pseudo-second-order model is depicted as follows [27]:

(2) $$Q_{t}=\frac{k_{2}Q_{e}^{2}t}{1+k_{2}Q_{e}t}$$

where *Q*_e_ and *Q*_t_ (mg g^−1^) are the number of ART molecules adsorbed onto the adsorbent at equilibrium and time *t*, respectively. *k*_1_ (min^−1^) and *k*_2_ (min^−1^) are the rate constants of the pseudo-first-order model and pseudo-second-order model, respectively.

Table 1 lists the relevant parameters of the pseudo-first-order and pseudo-second-order rate equations of the PPIM and PPNM. It can be seen from the table data that the second-order kinetic model was in good agreement (*R*^2^ > 0.99) with the kinetic adsorption data of ART on the PPIM/PPNM. The results suggested that the pseudo-second-order kinetic model was better for predicting the adsorption behavior of ART on the PPIM and PPNM, indicating that the chemical process may be the rate-limiting step in the adsorption process. This also indicated that the adsorption of the membrane included physical diffusion and chemical adsorption. 

**Figure 8:**
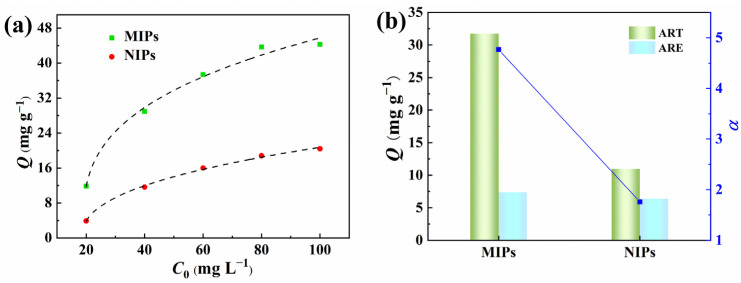
Adsorption isotherms of ART on MIPs and NIPs (**a**) and the selectivity separation factor (*α*) of MIPs/NIPs towards ART and ARE (**b**).

In order to verify the imprinting effects of the PPIM resulting from molecularly imprinted polymers, we investigated the binding isotherm and competitive binding of pre-synthesized MIP microspheres with ART and ARE. As depicted in Figure 8, the static equilibrium binding capacity of ART to MIPs initially increased rapidly as the initial ART concentration rose, and then reached a plateau. In contrast, the adsorption capacity of ART onto NIPs gradually increased with higher initial concentrations. It is evident that MIPs exhibited a substantially greater adsorption capacity for ART compared to NIPs, suggesting that MIPs possessed a larger number of specific binding sites for ART, enhancing the adsorption capacity. Furthermore, competitive adsorption experiments were conducted for coexisting compounds (ART and ARE) on both MIPs and NIPs at a concentration of 80 mg L^−1^. The *α*_ART/ARE_ value of MIPs for ART relative to ARE is 4.75, significantly exceeding that of NIPs, indicating that MIPs have a notably higher recognition ability for ART than NIPs.

**Figure 9:**
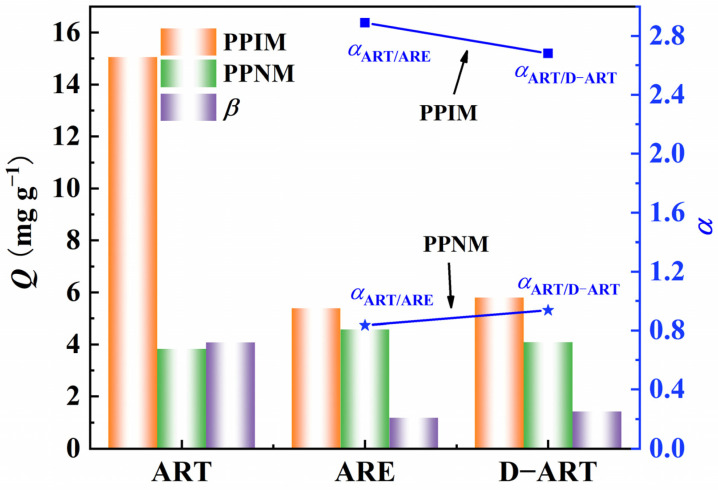
Selective adsorption abilities of PPIM/PPNM and selective factor (*β*) of PPIM toward PPNM as well as the square and star points stand for the selectivity factor (*α*) for PPIM (squares) and PPNM (stars) toward ART/ARE and ART/D−ART.

The selective adsorption capacity of the PPIM/PPNM was also investigated by evaluating their ability to adsorb ART toward the analogs of ARE and D−ART (Figure S4), which have similar, but different, molecular structures to ART. The adsorption concentrations of ART, ARE, and D−ART were fixed at 300 mg L^−1^. Figure 9 indicates that the PPIM showed a much higher adsorption ability toward ART and was comparatively insensitive to ARE or D−ART. The ART selective factor (*β*) of the PPIM toward PPNM reached 4.0, while those for ARE and D−ART were less than 1.20. Moreover, the selective rebinding factors of *α*_ART/ARE_ and *α*_ART/D-ART_ for the PPIM were 2.89 and 2.68, respectively, and were 0.835 and 0.947 for the PPNM, respectively. These results showed that the great template-imprinted effects of PPIM could be achieved via the imprinting process as it creates lots of artemisinin-imprinted sites.

### 2.9. Dynamic Adsorption Experiment

#### 2.9.1. Crossflow Adsorption of Different Flow Rates

**Figure 10:**
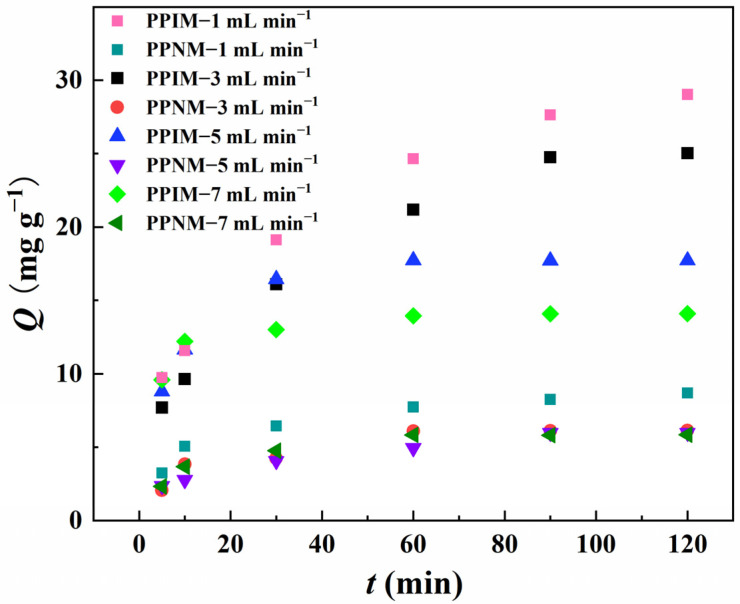
Dynamic adsorption of PPIM and PPNM toward ART at different flow rates of 1 mL min ^−1^, 3 mL min^−1^, 5 mL min^−1^ and 7 mL min^−1^.

The influences of different flow rate conditions (1 mL min^−1^, 3 mL min^−1^, 5 mL min^−1^, and 7 mL min^−1^) on the adsorption effect of the PPIM were studied and evaluated in detail, and the best dynamic adsorption conditions were obtained. In Figure 10, compared with the flow rates of 3 mL min^−1^, 5 mL min^−1^, and 7 mL min^−1^, the adsorption effect of the membrane was the best when the flow rate was 1 mL min^−1^, and its maximum adsorption capacity was 29.023, 25.02, 17.72, and 14.09 mg g^−1^, respectively. This may be because the membrane has a longer contact time with the target molecule under the condition of a low flow rate (1 mL min^−1^) to achieve more effective recognition. However, at the same time, the contact time between the PPNM and ART in the dynamic adsorption process was also longer, which may increase non-specific adsorption and decrease the selectivity. So, it is clear that the adsorption capacity of the PPNM toward ART at the flow rate of 1 mL min^−1^ was higher than that of the other flow rates. In consideration of practical industrial applications, the goal is to achieve rapid and efficient selective separation. Therefore, in the following experiment, we set the flow rate at 3 mL min^−1^ as the optimal separation rate.

#### 2.9.2. Crossflow Selective Separation

**Figure 11:**
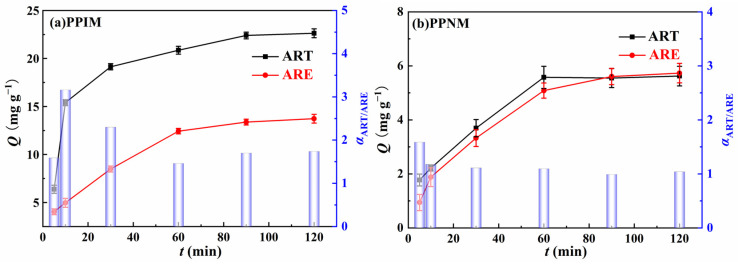
The adsorption capacity (*Q*_t_) (Point−line) of PPIM (**a**) and PPNM (**b**) toward ART and ARE, respectively, at different instants of time, as well as the selective separation factor (*α*_ART/ARE_) values (Columnar graphics) of PPIM (**a**) and PPNM (**b**).

The dynamic separation study was carried at a flow rate of 3 mL min^−1^ to evaluate the selective binding properties of the PPIM towards the binary component of ART and ARE with the mixed solution concentration of 300 mg L^−1^. Figure 11 shows the adsorption capacity of the membrane and the value of the selective separation coefficient *α* at different times. As shown in the figure, the adsorption capacity of the PPIM and PPNM on ART and ARE first increased rapidly, and then gradually stabilized. Compared with the PPNM, the PPIM had a larger adsorption capacity, which proved the existence of the imprinting effect. In addition, the selectivity factor of the PPIM for ART and ARE was 3.16 at 10 min, higher than that of the PPNM (ca. 1.5). These findings could be ascribed to the fact that more cavities or adsorption sites were obtained on the interior of the PPIM compared to the PPNM. After 30 min, the *α* decreased to about 1.5, indicating the weak separation ability. This may be ascribed to the effective sites being saturated by the ART molecules during the initial 30 min, so the separation ability became weaker. Therefore, it can be concluded that the PPIM has a good separation effect toward ART and ARE during the first 30 min.

Table 2 shows the comparison of the fluxes (*J*) and the selectivity between the PPIM in this work and the separation materials in previous research [28,29,30,31,32]. The membrane flux (800 L/m^2^/h) of the PPIM was much higher than most of the imprinted membranes, as was the selective separation factor (*α*_ART/ARE_). Thus, the as-prepared PPIM with better ART/ARE separating properties and the unique characteristics of 3D porous membranes exhibited good potential for practical applications in industry.

### 2.10. Reusability

**Figure 12:**
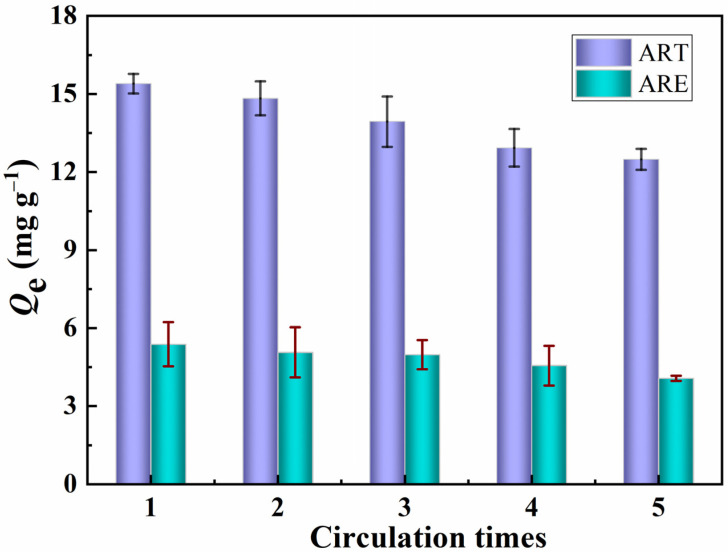
Regeneration performance of the PPIM toward ART and ARE.

The dynamic crossflow selective separation membrane was directly used in regeneration experiments to evaluate the stability and practicability of the PPIM. The adsorbed PPIM was eluted with CH_3_OH/HAc (*v*/*v*, 9:1) solution, and the adsorbed template molecule ART was removed and then used again for selective separation experiments. This operation was repeated five times. Figure 10 shows the adsorption capacity of the PPIM for ART and ARE. It can be seen from Figure 12 that the prepared membrane still maintains a relatively high adsorption and separation capacity (17% lower than the initial binding capacity) after being recycled five times, showing that the PPIM has relatively good regeneration performance.

## 3. Experimental Section

### 3.1. Apparatus

A water bath oscillator was used for the synthesis of the imprinted polymers under set temperature and speed (SHA-B). All particles were dried in a vacuum drying oven (HD-E804-55A, Kunshan Haida Precision Instrument Co., Ltd., Suzhou, China). An AHT-7700 transmission electron microscope (TEM, Hitachi, Japan) was employed to examine the morphologies of the synthesized polymers. The Fourier-transform infrared (FT-IR) spectra of the products were recorded using an FT-IR spectrometer (FTIR-2000, PerkinElmer, Inc., Shelton, CT, USA). The pore size distribution of the membrane was tested using a membrane pore size analyzer (PSDA-30, Nanjing Gaoqian Functional Material Technology Co., Ltd., Nanjing, China). The HPLC analysis was performed on an Agilent 1100 series with a Sharpsil-T-C18 column (4.6 × 250 mm, 5 μm, Sepax Technologies, Inc., Suzhou, China) at 30 °C. The mobile phase was composed of acetic acid aqueous solution/acetonitrile (40/60, *v*/*v*) at a flow rate of 1.0 mL/min. The detector wavelength was set at 213 nm for all analytes. 

### 3.2. Reagents and Materials

Polyvinylidene fluoride powder (PVDF, Mn¼ 110,000 g·mol^−1^; Sinopharm Chemical Reagent, Beijing, China), artemisinin (ART, 98%), artesunate (ARU, 98%), artemether (ARE, 98%), dihydroartemisinin (D-ART, 98%) were all purchased from Aladdin Co., Ltd. (Shanghai, China). Ethylene glycol dimethacrylate (EGDMA, 98%), 2,2-Azobisisobutyronitrile (AIBN, 98%), acrylamide (AM, 99%), acetonitrile (CH_3_CN, 98%), ethanol (C_2_H_5_OH, 99.5%), methyl alcohol (CH_3_OH, 99.5%), acetic acid (CH_3_COOH, 99.5%), and sodium chloride (NaCl, 99.5%) were all purchased from Sinopharm Chemical Reagent (Shanghai, China). All solvents used in the synthetic process were of at least analytical grade.

### 3.3. Synthesis of MIPs

In order to achieve an efficient polymer structure for imprinting ART, the monomer (AM, 0.0284 g) and cross-linkers (EGDMA, 0.0396 g) were chosen to prepare molecularly imprinted polymers (MIPs) via precipitation polymerization. Briefly, certain amounts of AM as a functional monomer (0.0284 g) and ARU as a dummy template (0.0384 g) were dissolved in 10 mL of acetonitrile and stored at 5 °C for 4 h. Then, the cross-linker agent of EGDMA (0.396 g), AIBN as an initiator (0.015 g), and 50 mL of acetonitrile were added to the above mixture and were uniformly dissolved together via sonication and purged with N_2_ gas (for 10 min) to remove the dissolved oxygen. The reaction vessel was sealed and placed in a water bath oscillator at 65 °C and reacted for 16 h. After polymerization, in order to remove the template and also to form specific cavities, the resultant products were collected and refluxed with an extraction solvent of methanol/acetic acid (9:1, *v*/*v*) in a Soxhlet for 24 h. Then, the polymers were washed several times with double distilled water and ethanol and then dried in a vacuum drying oven at 50 °C for 24 h. The non-imprinted polymers (NIPs) were synthesized using the same procedure but in the absence of a dummy template.

### 3.4. Preparation of Foam PVDF/PDA-Based Imprinted Membrane (PPIM) 

PVDF powder and sodium chloride (NaCl) particles were heated at 200 °C for 30 min to prepare the foam PVDF membrane according to the mass ratio *m*_PVDF_:*m*_NaCl_ = 1:6, 1:7, and 1:8. After that, the foam PVDF membrane was soaked in boiling water for 24 h to dissolve the NaCl using the sacrificial template. Finally, it was dried in a vacuum drying oven to obtain the porous foam PVDF membrane.

Then, 0.1 g of “Tris” buffer reagent was weighed into a 250 mL beaker, and then 100 mL of deionized water was added and the pH was adjusted to 8.5 using hydrochloric acid. Then, 0.2 g of dopamine hydrochloride, porous foam PVDF membrane, and 0.2 g of MIPs were added into the solution using sonication for 10 min. Then, the 250 mL beaker was stirred on a magnetic stirrer for 10 h. After the reaction, the porous foam PVDF membrane that had adhered to the PDA/MIPs was taken out, and the ionic water was replaced many times via sonication until the liquid in the beaker was colorless and clear. Finally, the foam PVDF/PDA-based imprinted membrane (PPIM) was taken out and placed in an electrothermal blast drying oven at 40 °C to dry. In order to calculate the amount of MIPs adhered to the foam PVDF membrane, the foam PVDF/PDA membrane was obtained the same way, but in the absence of dummy MIPs. The difference between the mass of the PPIM and the foam PVDF/PDA membrane is the amount of MIPs in the adhesion process. The foam PVDF/PDA-based non-imprinted membrane (PPNM) was synthesized the same way as the PPIM, except that NIPs were used instead of MIPs during the preparation process.

### 3.5. Characterization

A scanning electron microscope (SEM, S-4800, Tokyo, Japan) was used to observe the morphology of the MIPs and the microstructure of the membranes. The successful synthesis of the PPIM was confirmed using attenuated total reflectance Fourier-transform infrared spectroscopy (ATR-FTIR, Nicolet iS50, Waltham, MA, USA). AUTM (Topac 2000, Yeonjin Corporation, Seoul, Republic of Korea) was used to measure the mechanical properties at a crosshead speed of 10 mm min^−1^. In addition, the pore size distribution, flux, and contact angle of the membrane were also analyzed. HPLC is used to determine the concentration of ART and ARE to calculate the adsorption capacity. 

### 3.6. Static Adsorption Experiments

To explore the relationship between adsorption time and adsorption capacity, the following adsorption kinetic experiments were carried out. A piece of PPIM or PPNM was placed in a beaker containing 150 mL of 300 mg L^−1^ of ART ethanol solution. Samples were taken at different time intervals (5, 10, 30, 60, 120, 180, and 360 min), and finally, the concentration of the ART solution was detected using HPLC. The adsorption capacity of ART can be expressed using the following formula (Equation (3)):

*Q*_e_ = (*C*_o_ − *C*_e_) *V*/*M* (3)

where *Q*_e_ (mg g^−1^) is the adsorption capacity of the PPIM/PPNM, *C*_o_ and *C*_e_ are the initial and equilibrium concentrations (mg L^−1^), respectively, and *V* (L) and *M* (g) are the solution volume and adsorbent mass.

### 3.7. Dynamic Adsorption Experiment

#### 3.7.1. Crossflow Separation Experiment

Considering the actual application of the membrane, we placed the PPIM in a crossflow machine that was rinsed using absolute ethanol before use to study the effect of different flow rates on the adsorption performance of the PPIM. The specific operation was as follows: 150 mL of adsorption solution (ART in ethanol) was added to the storage tank at a temperature of 25 °C, and the flow rates of the three parallel experiments were 3, 5, and 7 mL min^−1^, respectively. After starting the experiment, samples were taken at 0, 5, 10, 30, 60, 120, 180, and 240 min, and the concentration of the samples was detected using HPLC. Then, the adsorption capacity was calculated using Equation (3).

#### 3.7.2. Crossflow Selective Separation Experiment

Since the selectivity of the PPIM is very important for the separation and selective recognition of ART, we conducted selective experiments in a mixed solution of ART and ARE with similar structures. The process was as follows: the membrane (PPIM or PPNM) was fixed onto the support screen (12.56 cm^−2^ effective filtration area) of the low-pressure crossflow experimental equipment (Figure S1). Then, 150 mg L^−1^ ART ethanol feed solution was added to the material tank at a certain dynamic flow rate (3.0 mL min^−1^) at 25 °C under normal pressure. Samples were taken at different times (0, 5, 10, 30, 60, 120, 180, and 240 min), and the concentration of ART in the adsorption solution was detected using HPLC. The selectivity factor can be calculated using the following equation (Equation (4)):

*α* = (*C*_pt/_*C*_ft_) (4)

where *C*_pt_ and *C*_ft_ represent the percentage mass of ART and ARE collected in the permeate and feed streams, respectively.

#### 3.7.3. Reusability Experiments

The recyclability of the membrane is also an important indicator reflecting its performance. In this experiment, the PPIM was subjected to five cycles of adsorption, desorption, and re-adsorption to explore the regeneration capacity. The specific operation was the same as the crossflow selective separation experiment, except that the membrane was desorbed when it reached the adsorption equilibrium and then recycled (methanol/acetic acid mixture 90:10, *v*/*v*).

## 4. Conclusions

In summary, a 3D porous foam membrane (PPIM) was successfully prepared by introducing pre-synthesized MIPs into the interior of a foam PVDF membrane via dopamine adhesion, which can avoid the defects of recognition site encapsulation. The preparation method is simple in operation, low in cost, and environmentally friendly. Moreover, the 3D-type foam PPIM possessed a relatively large flux, which is beneficial for industrial separation applications. In addition, the pseudo-second-order rate equation described the kinetic curve well. Moreover, the foam membrane can be used at least five times without significant weakening of the binding ability. Compared with non-imprinted foam (PPNM), imprinted foam (PPIM) has significant adsorption performance, whereas non-imprinted foam exhibits poor non-specific binding. The dynamic crossflow selective separation research showed that the selective separation ability (*a*) of the PPIM towards ART/ARE reached its peak value (3.16) within just 10 min, which suggests that the high flux of PPIM can increase the separation efficiency. This research provides an effective and simple method for preparing MIMs for the medical extraction of ART.

## Figures and Tables

**Table 1:** The regression parameters of the pseudo−first−order kinetic equation and pseudo−second−order kinetic equation.

	Pseudo−First−Order Kinetic Equation			Pseudo−Second−Order Kinetic Equation		
	*k*_1_ (min^−1^)	*Q*_e_ (mg g^−1^)	*R* ^2^	*k*_2_ (min^−1^)	*Q*_e_ (mg g^−1^)	*R* ^2^
PPIM	0.06693	13.9973	0.9257	1.7170	15.0550	0.9963
PPNM	0.07657	3.6293	0.9780	0.1923	3.8327	0.9958

**Table 2:** Selective adsorption parameters of PPIM and other reported MIPs/MIM.

ART−Imprinted Polymers/Membrane (MIM/MIPs)	*J* (L/m^2^/h)	Selective Separation Factor (*α*_ART/ARE_)	Ref.
PPIM	800	3.16	This work
MIM	0.4436	2.15	[28]
MIM	0.0076	2.939	[29]
SPIMs	0.0418	2.76	[30]
MIPs	——	2.88	[31]
MIPs	——	2.27	[32]

## Supplementary Materials

The following supporting information can be downloaded at: https://www.mdpi.com/article/10.3390/molecules28217452/s1, Figure S1: Physical image of low-pressure flat membrane cross-flow device with front view (a) and top view (b); Figure S2: The physical map and SEM images of the three different foam PVDF membrane with the varied dosage of PVDF powder and sodium chloride particles (the mass ratios were 1:6 (a), 1:7 (b) and 1:8 (c)). Figure S3. SEM surface images of PPIM (a,b) and PPNM (c,d) with the different magnifications. Figure S4. The molecular structure of ART and ARE and D-ART.
