# Supplementary material for: Three-Dimensional Porous PVDF Foam Imprinted Membranes with High Flux and Selectivity toward Artemisinin/Artemether
Source: Molecules. 2023 Nov 6;28(21):7452. doi: 10.3390/molecules28217452 (PMC10647727; doi:10.3390/molecules28217452)
Supplement: Supplementary file 1 [file molecules-28-07452-s001.zip › molecules-2594943-SI.pdf]

# Three-Dimensional Porous PVDF Foam Imprinted Membranes with High Flux and Selectivity toward Artemisinin/Artemether

**Weibai Bian [1,2,3,*], Ruixuan Zhang [1], Xiaohui Chen [1], Chuanxun Zhang [2] and Minjia Meng [2,*]**

1 School of Photoelectric Engineering, Changzhou Institute of Technology, Changzhou 213032, China
2 School of Chemistry and Chemical Engineering, Jiangsu University, Zhenjiang 212013, China
3 Tianhe Pharmaceutical Co., Ltd., Yangzhou 225267, China
* Correspondence: bianwb@czu.cn (W.B.); mmj@ujs.edu.cn (M.M.)

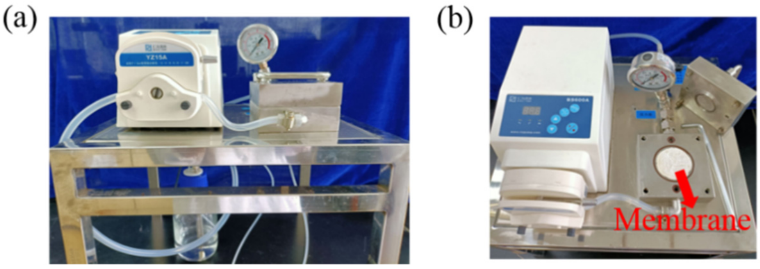


**Figure S1.** Physical image of low-pressure flat membrane cross-flow device with front view (a) and top view (b).

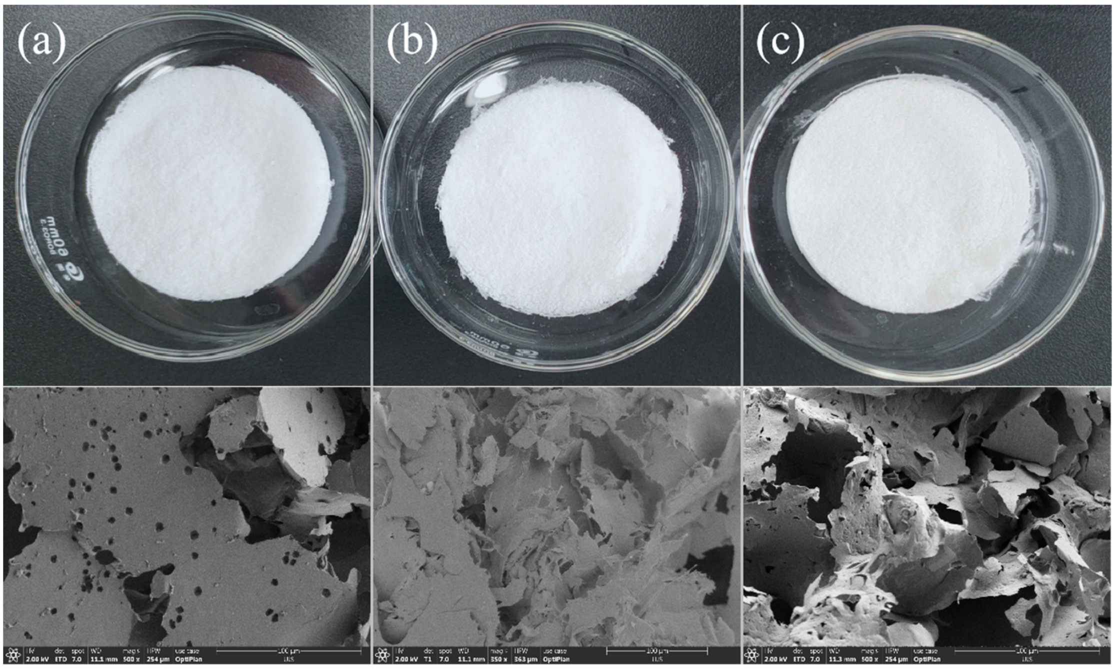


**Figure S2.** The physical map and SEM images of the three different foam PVDF membrane with the varied dosage of PVDF powder and sodium chloride particles (the mass ratios were 1:6 (a), 1:7 (b) and 1:8 (c)).

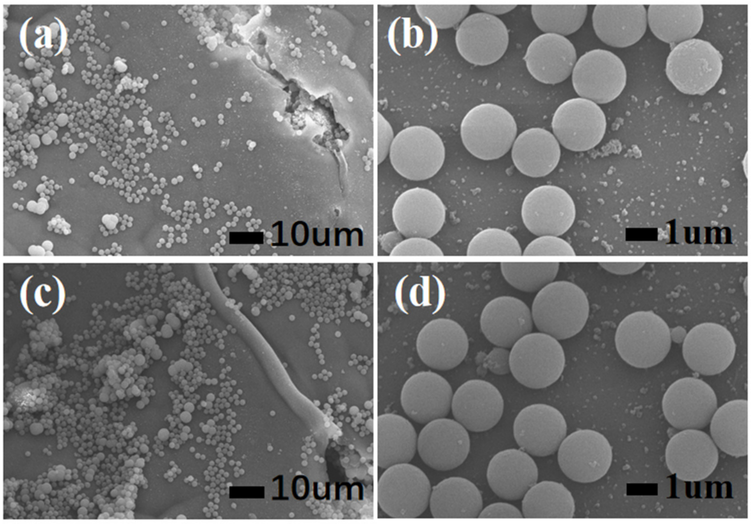


**Figure S3.** SEM surface images of PPIM (a,b) and PPNM(c,d) with the different magnifications.

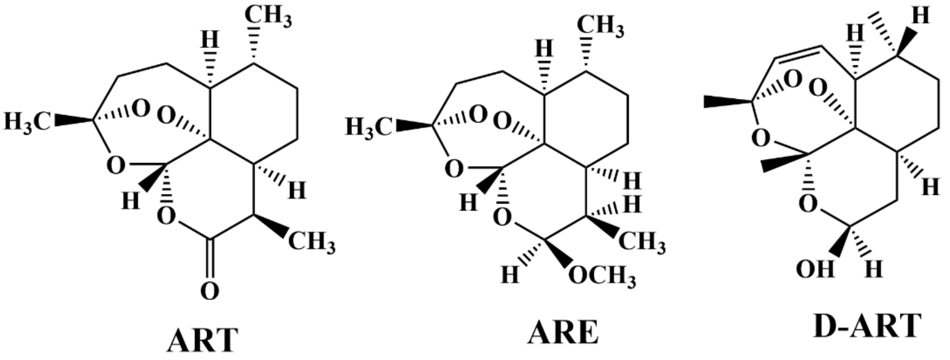


**Figure S4.** The molecular structure of ART and ARE and D-ART
